# Supplementary material for: Potentiating antibiotic efficacy via perturbation of non-essential gene expression
Source: Commun Biol. 2021 Nov 5;4:1267. doi: 10.1038/s42003-021-02783-x (PMC8571399; doi:10.1038/s42003-021-02783-x)
Supplement: Supplementary file 2 — Supplementary Information [file 42003_2021_2783_MOESM2_ESM.pdf]

Supporting Online Material for

Potentiating antibiotic efficacy via perturbation of non-essential gene expression

Peter B. Otoupal[1,2,3#], Kristen A. Eller[1#], Keesha E. Erickson[1], Jocelyn Campos[1], Thomas R. Aunins[1], and Anushree Chatterjee[1,4,5,6*].

Affiliations:
[1]Department of Chemical and Biological Engineering, University of Colorado at Boulder, Boulder, CO 80303, USA.
[2]Joint Bioenergy Institute, Lawrence Berkeley National Laboratory, Emeryville, CA, 94608, USA
[3]Biomass Science and Conversion Technology Department, Sandia National Laboratories, Livermore, CA, 94551, USA
[4]Biomedical Engineering, University of Colorado at Boulder, Boulder, CO, 80303, USA
[5]Sachi Bioworks, Inc, Boulder, CO, 80301, USA
[6]Antimicrobial Regeneration Consortium, Boulder, CO, 80301, USA

# These authors contributed equally

*Address correspondence to: Dr. Anushree Chatterjee (chatterjee@Colorado.EDU)

This PDF file includes:
Supplementary Figures 1-11
Supplementary Tables 1-4

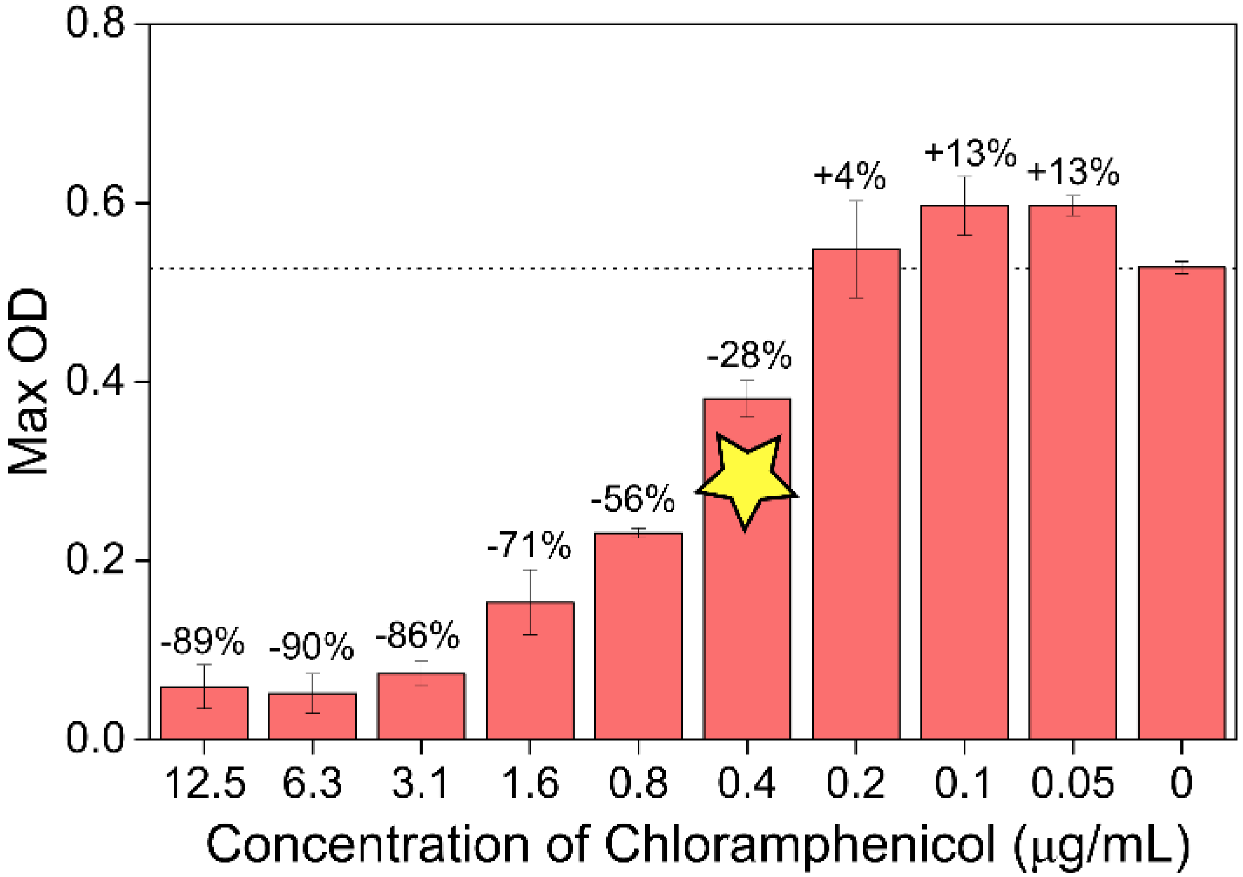

Supplementary Figure 1. Example of an antibiotic dilution test to identify the drug concentration suitable for combination therapy. *E. coli* BW25113 was grown in a range of concentrations for 16 hours. The concentration that resulted in a maximum optical density 10-50% lower than the no treatment case was selected for each antibiotic (indicated here with a star at 0.4 µg/mL of chloramphenicol). Error bars are SD, n = 3.

a

Gene	Nodes	Edges	Avg Node Degree	Local Clus. Coef.
ΔacrA	16	45	5.6	0.652
ΔbglG	19	71	7.5	0.801
Δcrp	15	32	4.3	0.856
ΔcsgD	8	28	7.0	1.000
ΔcyoA	42	396	18.9	0.821
Δdam	21	110	10.5	0.904
ΔdinB	16	30	3.8	0.865
Δfiu	17	77	9.1	0.921
ΔflhC	43	665	30.9	0.952
ΔflhD	44	646	29.4	0.945
Δfnr	10	16	3.2	0.919
Δfur	6	9	3.0	0.844
ΔgadX	19	102	10.7	0.853
Δhfq	20	75	7.5	0.866
ΔmarA	7	11	3.1	0.857
ΔmutS	33	128	7.8	0.724
ΔphoP	20	46	4.6	0.855
ΔpolB	15	40	5.3	0.837
ΔrecA	69	509	14.8	0.652
Δrob	19	70	7.4	0.941
ΔrpoS	28	109	7.8	0.786
ΔsoxS	8	10	2.5	0.810
Δtar	60	1189	39.6	0.882
ΔtolC	49	188	7.7	0.718
Δwzc	34	214	12.6	0.826
ΔybjG	8	10	2.5	0.851
ΔydhY	13	36	5.5	0.806
ΔydiV	45	307	13.6	0.712
ΔyehS	6	8	2.7	0.883
ΔyjjZ	55	540	19.6	0.740

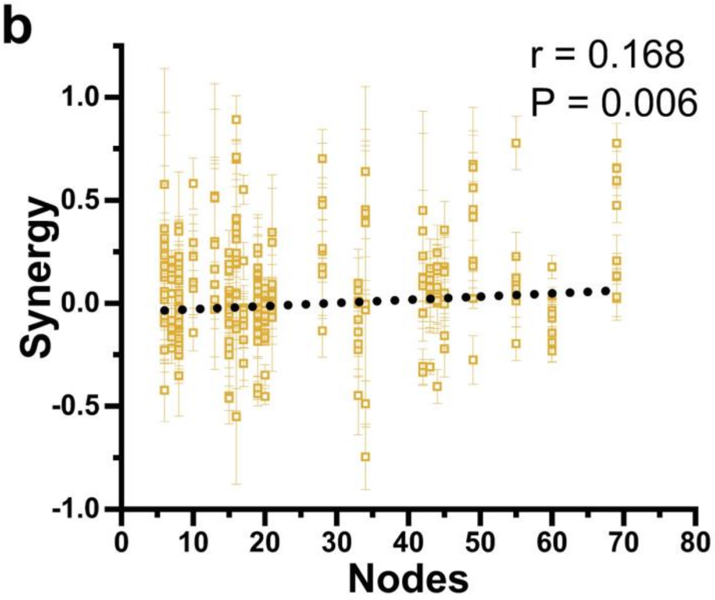

Supplementary Figure 2. The STRING database was used to collect information on all the known protein-protein interactions that each of the thirty gene knockouts are involved in *E. coli* MG1655 [1]. (**a**) Tabulated information on nodes (total amount of proteins in network), edges (total amount of protein-protein interactions in network), average node degree (average amount of interactions each protein is involved in), and local cluster coefficient (the "tightness" of the network indicating the degree of interaction between the network overall) for each gene knockout, with larger values as green and smaller values as red. (**b**) The number of nodes of each gene knockout was plotted against the degree of synergy they exhibited with each antibiotic (a total of 270 datapoints). A significant positive correlation was identified, and the Pearson's correlation coefficient and associated P-value are presented. Y-axis error bars indicate standard deviation of synergy derived from at least three biological replicates.

a

		Synergy			
		Gene Knockout		CRISPRi	
Ceft	ydhY	0.52 ± 0.55	Synergistic	-0.05 ± 0.11	Additive
	tolC	0.46 ± 0.38	Synergistic	0.03 ± 0.08	Additive
	phoP	0.01 ± 0.08	Additive	-0.01 ± 0.10	Additive
	soxS	0.23 ± 0.17	Synergistic	0.02 ± 0.10	Additive
	marA	0.21 ± 0.05	Synergistic	-0.01 ± 0.13	Additive
	crp	-0.05 ± 0.06	Additive	-0.11 ± 0.13	Antagonistic
Tet	rpoS	0.50 ± 0.22	Additive	-0.17 ± 0.09	Antagonistic
	**tolC**	**0.42 ± 0.31**	**Synergistic**	**0.49 ± 0.25**	**Synergistic**
	yehS	0.36 ± 0.45	Additive	-0.10 ± 0.08	Antagonistic
	**acrA**	**0.41 ± 0.06**	**Synergistic**	**0.62 ± 0.41**	**Synergistic**
	csgD	0.37 ± 0.26	Synergistic	-0.11 ± 0.28	Additive
	crp	0.19 ± 0.06	Synergistic	-0.12 ± 0.09	Antagonistic
Erm	**wzc**	**0.64 ± 0.41**	**Synergistic**	**0.10 ± 0.19**	**Synergistic**
	**cyoA**	**0.45 ± 0.48**	**Synergistic**	**0.08 ± 0.17**	**Synergistic**
	**acrA**	**0.19 ± 0.04**	**Synergistic**	**0.36 ± 0.16**	**Synergistic**
	**tolC**	**0.18 ± 0.03**	**Synergistic**	**0.36 ± 0.15**	**Synergistic**
	recA	0.13 ± 0.04	Synergistic	0.00 ± 0.18	Additive
	yehS	0.58 ± 0.56	Synergistic	0.02 ± 0.12	Additive
Pur	**tolC**	**0.67 ± 0.28**	**Synergistic**	**0.64 ± 0.34**	**Synergistic**
	**acrA**	**0.70 ± 0.10**	**Synergistic**	**0.69 ± 0.40**	**Synergistic**
	recA	0.65 ± 0.08	Synergistic	-0.16 ± 0.41	Additive
	ydhY	0.51 ± 0.43	Synergistic	-0.11 ± 0.43	Additive
	wzc	0.39 ± 0.35	Synergistic	-0.14 ± 0.39	Additive
	bglG	0.24 ± 0.08	Synergistic	0.00 ± 0.45	Additive
Cip	tolC	0.56 ± 0.25	Synergistic	0.02 ± 0.15	Additive
	**fnr**	**0.58 ± 0.12**	**Synergistic**	**0.96 ± 0.17**	**Synergistic**
	wzc	0.44 ± 0.33	Synergistic	0.08 ± 0.24	Additive
	fur	0.32 ± 0.12	Synergistic	0.04 ± 0.24	Additive
	**rpoS**	**0.17 ± 0.05**	**Synergistic**	**0.07 ± 0.15**	**Synergistic**
	**recA**	**0.21 ± 0.04**	**Synergistic**	**0.85 ± 0.18**	**Synergistic**
Trim	**acrA**	**0.71 ± 0.08**	**Synergistic**	**0.67 ± 0.18**	**Synergistic**
	**tolC**	**0.66 ± 0.07**	**Synergistic**	**0.60 ± 0.16**	**Synergistic**
	**recA**	**0.59 ± 0.07**	**Synergistic**	**0.06 ± 0.17**	**Synergistic**
	ydiV	0.36 ± 0.14	Synergistic	0.09 ± 0.28	Additive
	csgD	0.35 ± 0.07	Synergistic	-0.01 ± 0.13	Additive
	fnr	0.29 ± 0.14	Synergistic	-0.02 ± 0.13	Additive

b

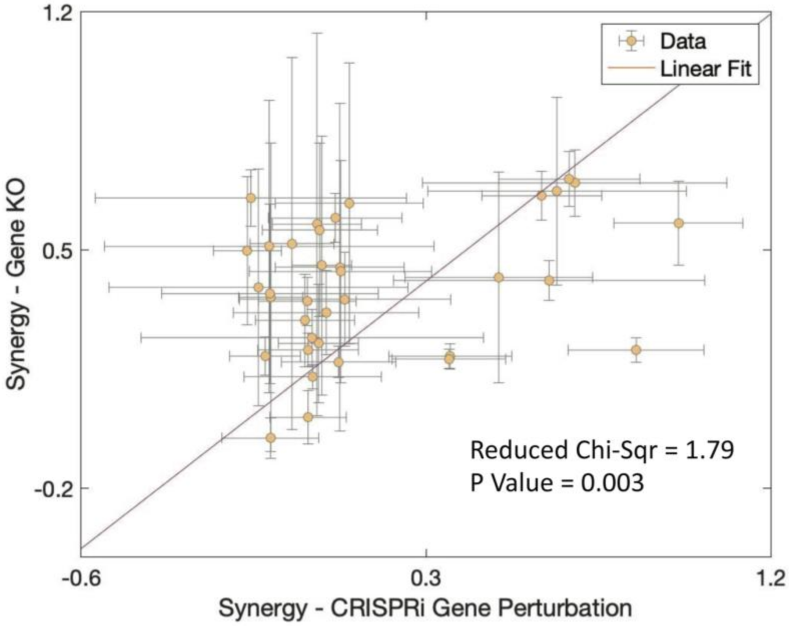

Supplementary Figure 3. Correlation between gene-drug synergies induced by gene knockout and gene perturbation with CRISPRi. (**a**) Raw tabulated data of all synergy values and their errors. Synergistic interactions are highlighted in red and antagonistic interactions are highlighted in green. Interactions where synergy was seen in both gene knockout and CRISPRi perturbations are highlighted across the entire row in grey and in bold. (b) The overall linear correlation of each of the 36 pairs of data from gene knockout and gene perturbation with CRISPRi, the respective reduced chi-sqr value and its significance value.

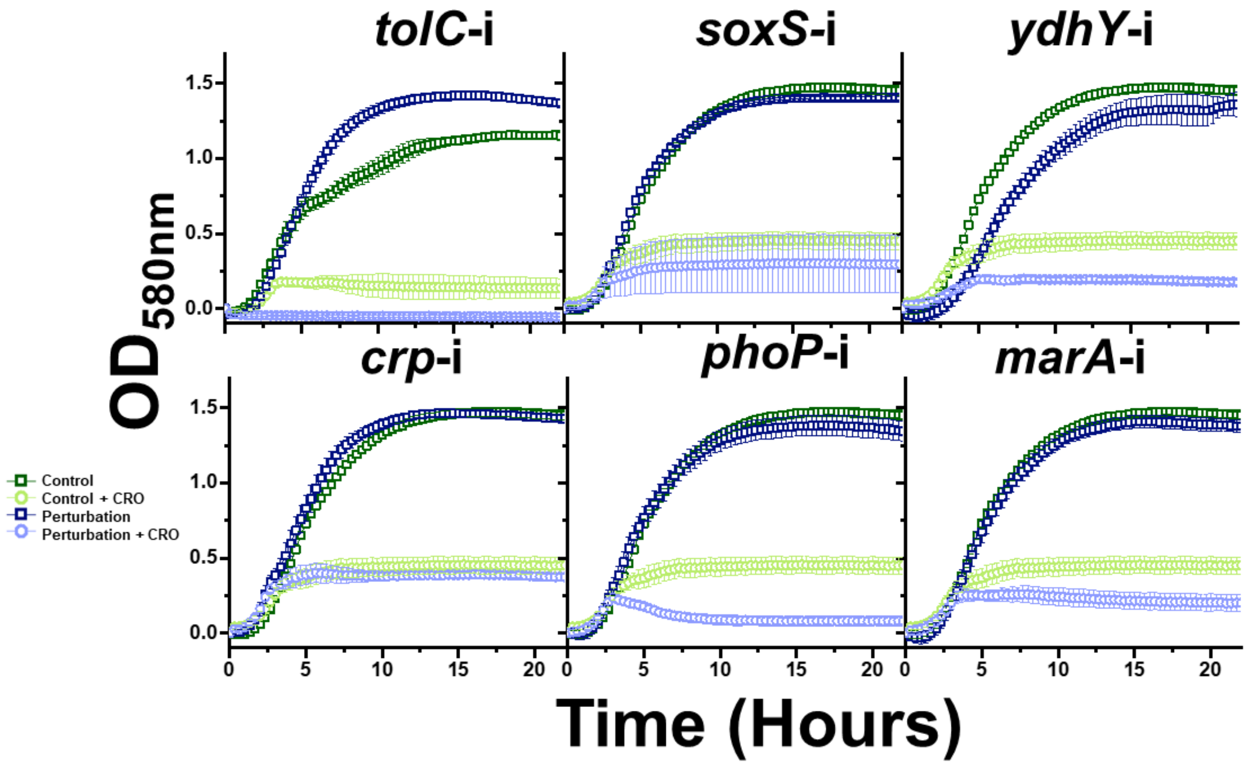

Supplementary Figure 4. Growth of CRISPRi strains during exposure to 2.0 µg/mL ceftriaxone in LB medium. Error bars represent standard deviation of four biological replicates. Growth is normalized to starting ODs. CRISPRi led to significantly lower final ODs in relation to the control perturbation with antibiotic when targeting *tolC*, *ydhY*, *phoP*, and *marA*.

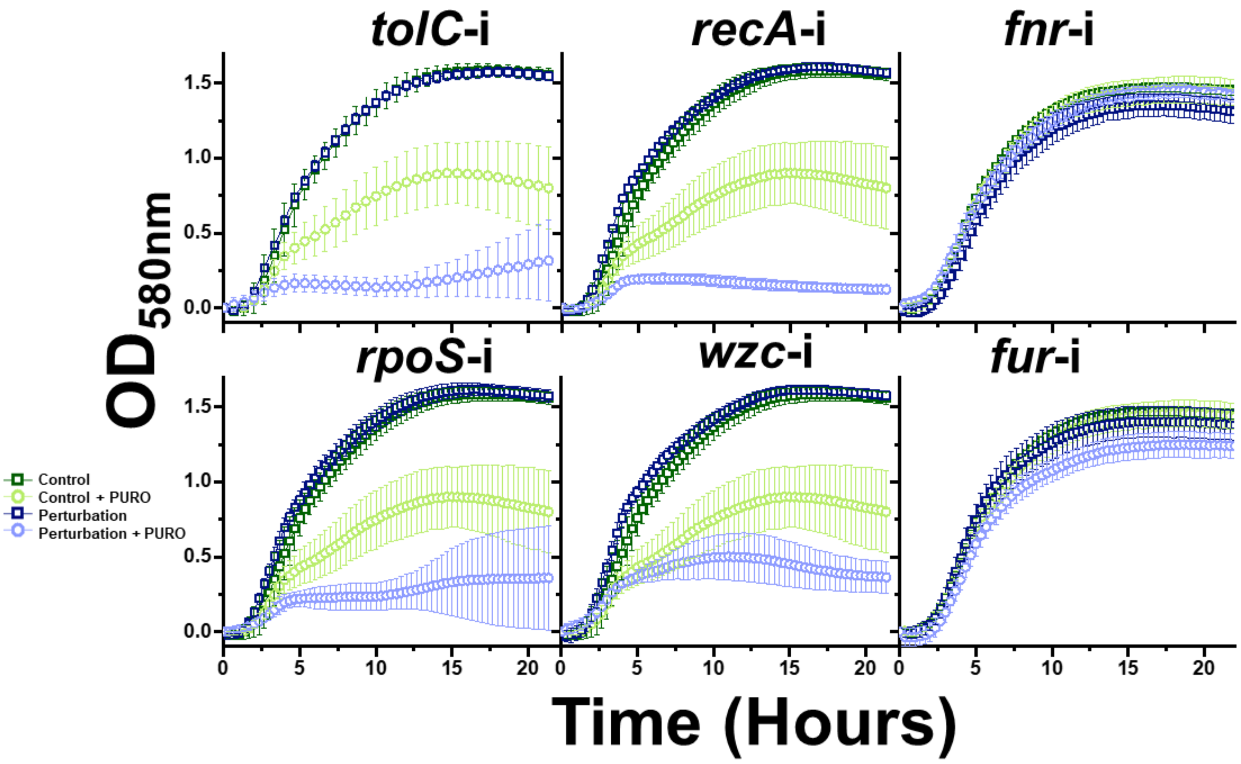

Supplementary Figure 5. Growth of CRISPRi strains during exposure to 50.0 µg/mL puromycin in LB medium. Error bars represent standard deviation of four biological replicates. Growth is normalized to starting ODs. CRISPRi led to significantly lower final ODs in relation to the control perturbation with antibiotic when targeting *tolC*, *recA*, *rpoS*, *wzc*, and *fur*.

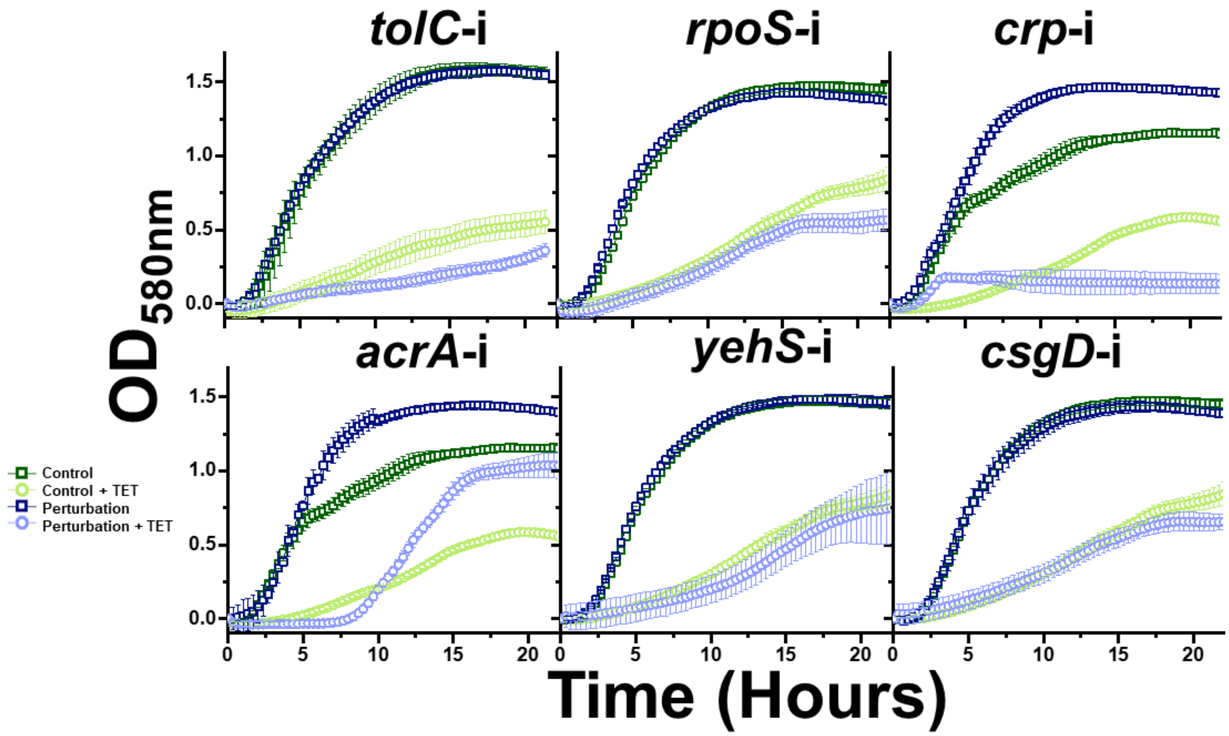

Supplementary Figure 6. Growth of CRISPRi strains during exposure to 0.25 µg/mL tetracycline in LB medium. Error bars represent standard deviation of four biological replicates. Growth is normalized to starting ODs. CRISPRi led to significantly lower final ODs in relation to the control perturbation with antibiotic when targeting *tolC*, *rpoS*, *crp*, and *csgD*.

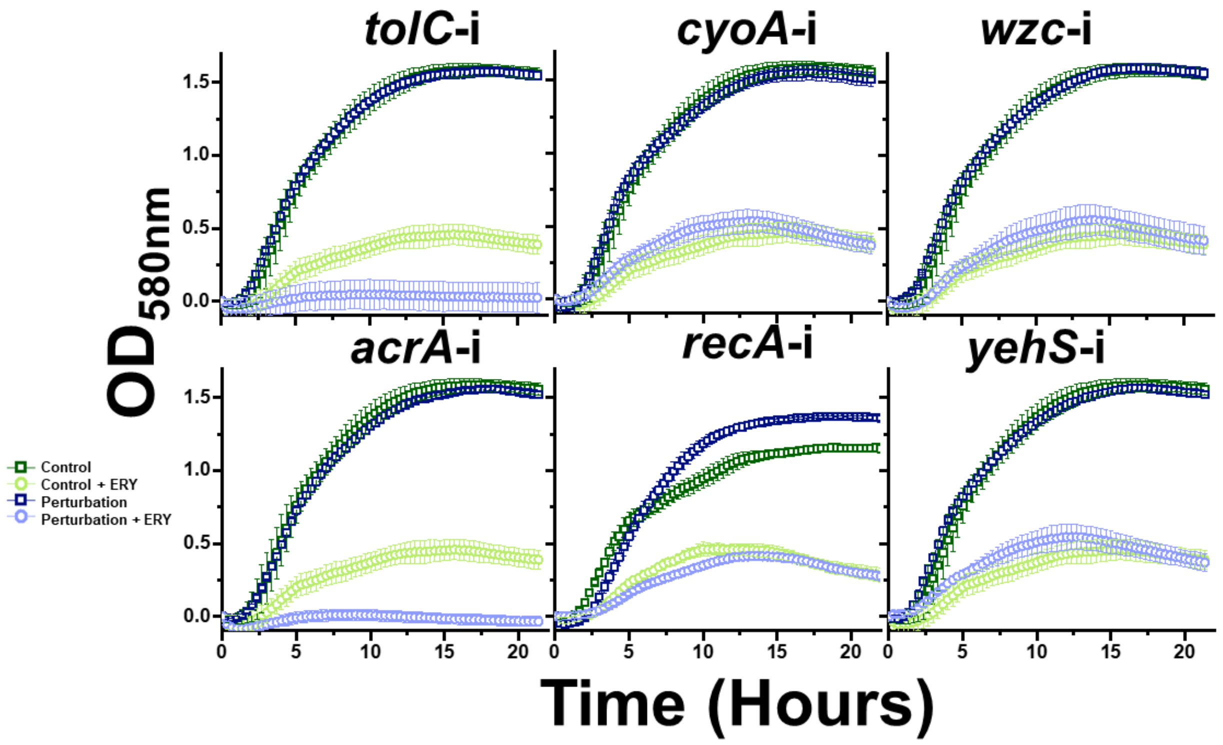

Supplementary Figure 7. Growth of CRISPRi strains during exposure to 50.0 μg/mL erythromycin in LB medium. Error bars represent standard deviation of four biological replicates. Growth is normalized to starting ODs. CRISPRi led to significantly lower final ODs in relation to the control perturbation with antibiotic when targeting *tolC* and *acrA*.

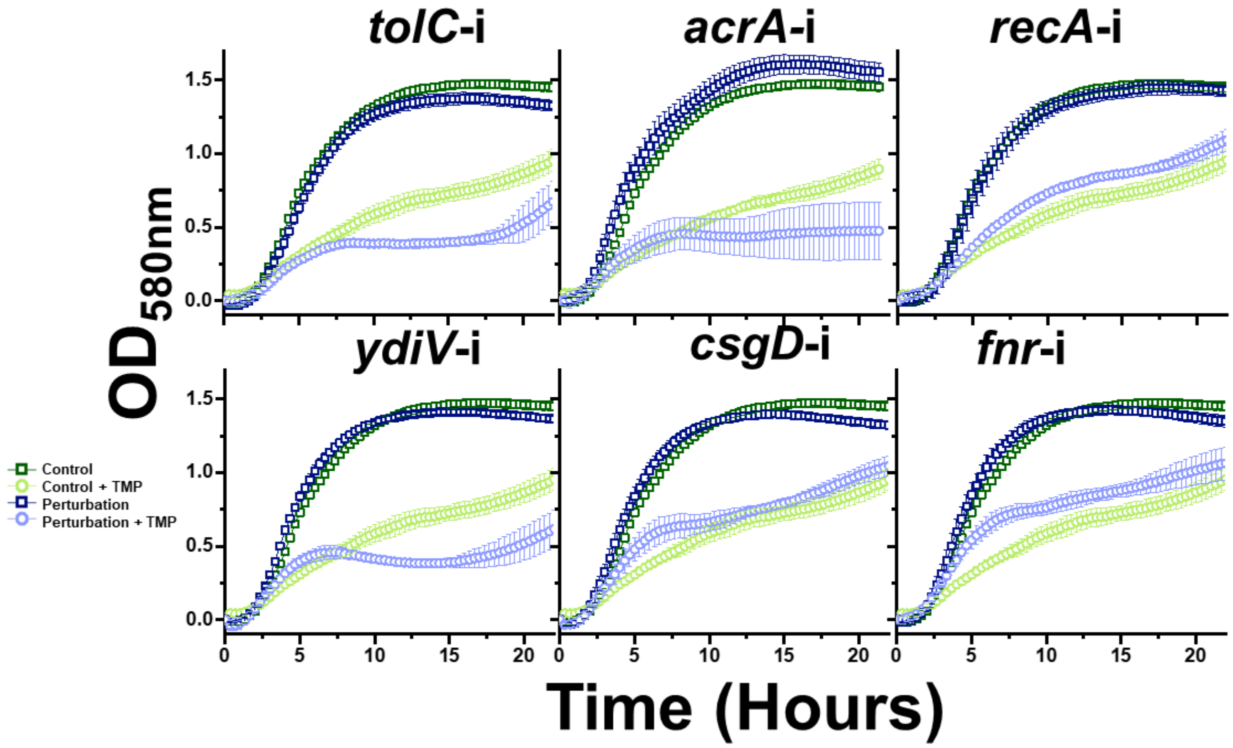

Supplementary Figure 8. Growth of CRISPRi strains during exposure to 0.125 μg/mL trimethoprim in LB medium. Error bars represent standard deviation of four biological replicates. Growth is normalized to starting ODs. CRISPRi led to significantly lower final ODs in relation to the control perturbation with antibiotic when targeting *tolC*, *acrA*, and *ydiV*.

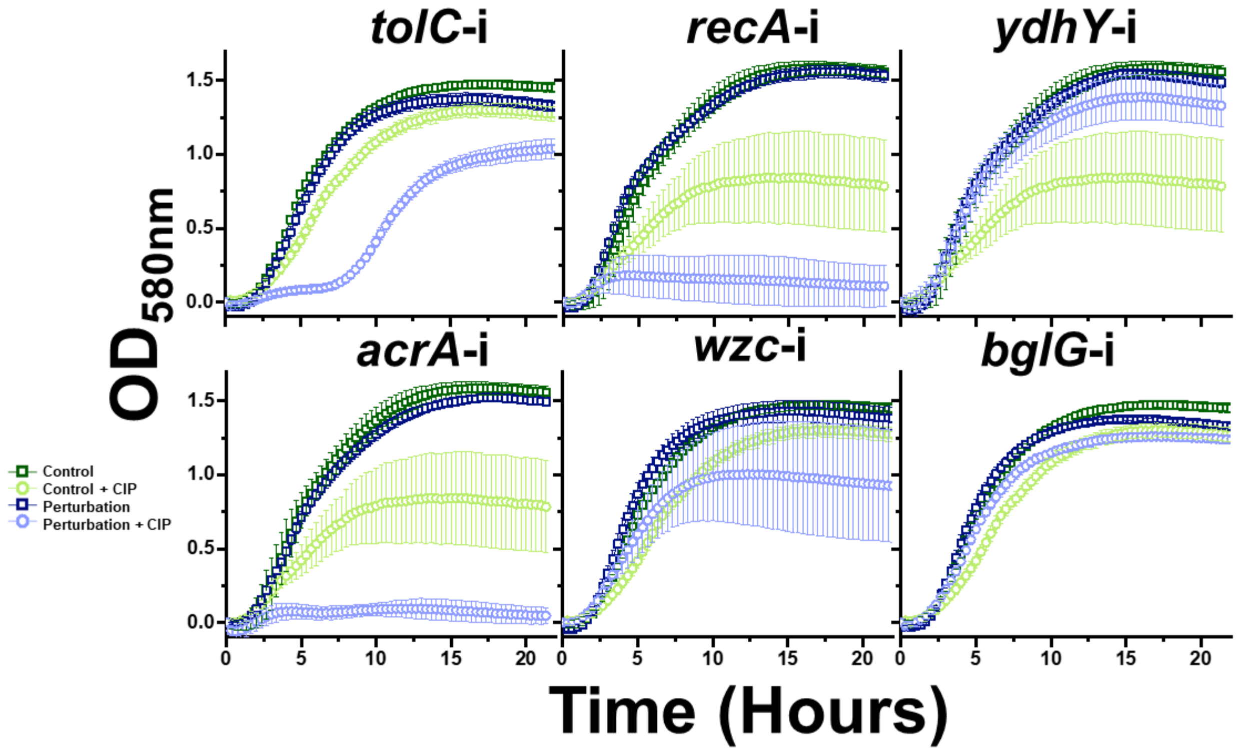

Supplementary Figure 9. Growth of CRISPRi strains during exposure to 0.008 µg/mL ciprofloxacin in LB medium. Error bars represent standard deviation of four biological replicates. Growth is normalized to starting ODs. CRISPRi led to significantly lower final ODs in relation to the control perturbation with antibiotic when targeting *tolC*, *recA*, and *acrA*.

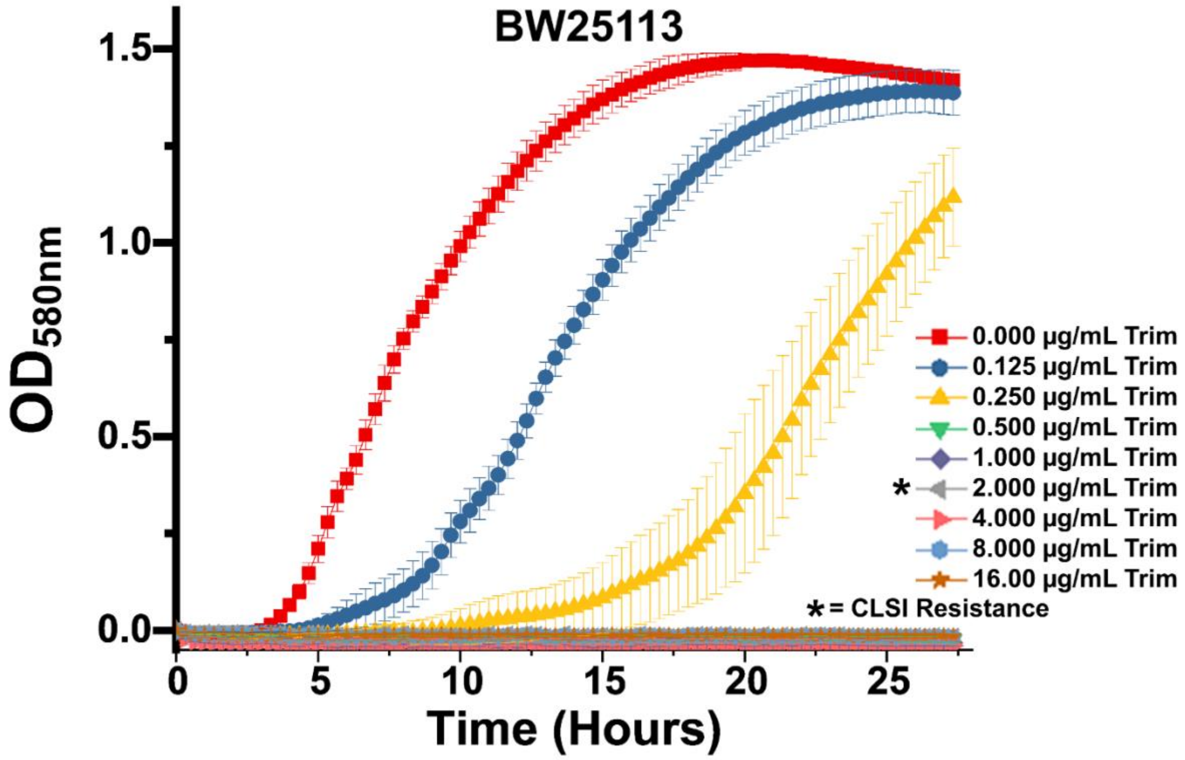

Supplementary Figure 10. BW25113 growth tests of trimethoprim resistance Cultures of BW25113 were grown in caMHB for 24 hours to quantify basal *E. coli* resistance to trimethoprim. Cells were unable to survive 0.5 µg/mL trimethoprim and above, 4-fold below the CLSI breakpoint for trimethoprim resistance. Error bars represent standard deviation of biological triplicates.

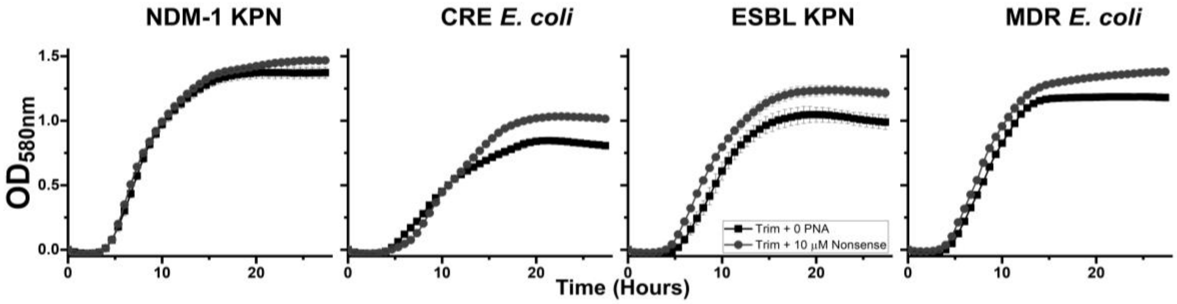

Supplementary Figure 11. Growth of clinically isolated MDR bacteria during exposure to 2.0 μg/mL trimethoprim in the presence or absence of 10 μM nonsense targeting PNA. Error bars represent standard deviation of biological triplicates.

Table S1. sgRNA targets examined in this study.

Target	Sequence
soxS	ctacatcaatgttaagcggc
tolC	ggctcaggccgataagaatg
acrA	agcatcagaacgaccgccag
ydhY	gatcgtccactattagatat
crp	aaacagacccgactctcgaa
phoP	agaaataaaaatgcgcgtac
marA	ccagtccaaaatgctatgaa
recA	taccaaattgtttctcaatc
wzc	caacatgccgctccggtaac
bglG	aattctcaacaataatgttg
rpoS	actgggttcctgttctacta
yehS	gcgtgcgctacattttgaaa
csgD	aatgaagtccatagtattca
fnr	tcccggaaaagcgaattata
fur	aataccgccctaaagaaagc
cyoA	aggaaatacaataaaagttt
ydiV	aatcagaatgataaagattc
rfp (nonsense control)	aactttcagtttagcggtct

Table S2. Potential off-targets of CRISPRi constructs. We screened the 9-11nt seed sequence of the sgRNAs from table S1 against the MG1655 genome for matching sequences indicating potential off-targets in any part of the indicated genes. For *rfp*-i, all matching off-targets are presented regardless of the adjacent PAM sequence. For all others, only sequences with a NGG PAM are reported below. Whether or not the deletion of each gene has been reported to impact fitness is also detailed using data from EcoCyc [2].

CRISPR target	Off-target	Seed length	PAM	Fitness impact when deleted?
rfp	*flgK*	9	GAC	None
rfp	*phoP*	9	GGC	None
rfp	*dbpA*	9	GGC	None
rfp	*rsuA/radA* (promoter)	9	AAT	None
rfp	*cysG*	9	CCT	No Growth – M9 gluc, MOPS gluc
rfp	*yiaT*	9	GGC	None
rfp	*glvC*	9	GAC	None
rfp	*frvR*	9	GTT	None
rfp	*ftsI*	9	TTA	No Growth – LB Lennox
rfp	*yfcC*	10	GGA	None
rfp	*acrD*	10	TGG	None
rfp	*ysgA*	10	GGT	None
rfp	*metL*	10	GCT	No Growth – M9 gluc/glyc, MOPS gluc
rfp	*ybhJ*	11	GTA	None
rfp	*yjiV*	11	CAG	None
soxS	*mntR*	9	NGG	None
soxS	*recF*	9	NGG	None
soxS	*ftsN* (promoter)	9	NGG	No Growth – LB Lenox
soxS	*fabR* (promoter)	9	NGG	None
tolC	*ncDNA 1*	9	NGG	N/A
tolC	*ncDNA 2*	9	NGG	N/A
tolC	*yejM*	9	NGG	No Growth – LB Lennox
acrA	*ttdT*	9	NGG	None
acrA	*dppB*	9	NGG	None
acrA	*yjhQ*	9	NGG	None
acrA	*phoB*	9	NGG	None
acrA	*torA*	9	NGG	None
acrA	*wbbK*	9	NGG	None
acrA	*yfaL*	9	NGG	None
acrA	*yfhM*	9	NGG	None
acrA	*hypF*	9	NGG	None
acrA	*mscM*	10	NGG	None
acrA	*ydbK*	10	NGG	None
acrA	*alaA*	11	NGG	None
ydhY	*pyrD*	9	NGG	No Growth – M9 gluc/glyc, MOPS gluc
crp	*leuS*	10	NGG	No Growth – LB Lenox
crp	*ycaC*	10	NGG	None
phoP	*argE*	9	NGG	No Growth – M9 gluc/glyc, MOPS gluc
phoP	*djlC*	9	NGG	None
phoP	*bioA*	9	NGG	No Growth – M9 gluc/glyc, MOPS gluc
phoP	*puuE*	9	NGG	None
phoP	*ycjR*	9	NGG	None
phoP	*pdxK*	9	NGG	None
phoP	*dnaB*	10	NGG	No Growth – LB Lennox
phoP	*cfa*	10	NGG	None
recA	*arnA*	9	NGG	None
wzc	*fryC*	9	NGG	None
wzc	*torC*	9	NGG	None
wzc	*mutS*	10	NGG	None
bglG	*ghoS*	9	NGG	None
bglG	*hyaB*	9	NGG	None
bglG	*cheY*	11	NGG	None

Table S2 Continued.

CRISPR target	Off-target	Seed length	PAM	Fitness impact when deleted?
yehS	*bdcA*	9	NGG	None
yehS	*tsx*	9	NGG	None
yehS	*yehL*	9	NGG	None
yehS	*yfdX* (promoter)	10	NGG	None
yehS	*ygeI*	10	NGG	None
yehS	*yobB* (promoter)	10	NGG	None
fnr	*nfrA*	10	NGG	None
fur	*arrS*	9	NGG	N/A
fur	*cutA* (promoter)	9	NGG	None
fur	*yaeF*	9	NGG	None
fur	*dgcC*	9	NGG	None
fur	*abrB* (promoter)	10	NGG	None
fur	*etk*	10	NGG	None
fur	*kch*	10	NGG	None
fur	*ydbA* (promoter)	10	NGG	None
cyoA	*arpA* (promoter)	9	NGG	None
cyoA	*udic*	10	NGG	None

Table S3. PNA targets examined in this study.

Target	Sequence
AcrA	KFFKFFKFFK-O-tatgtaaacctc
Fnr	KFFKFFKFFK-O-gatcataggtct
CsgD	KFFKFFKFFK-O-tgatgaaacccc
RecA	KFFKFFKFFK-O-gtcgatagccat
Nonsense	KFFKFFKFFK-O-gaataagggcga

Table S4. Potential PNA off-targets. Off-targets were identified as having the same sequence within +/- 6 nts of the noted gene's translation start site. The potential off-targets for a hypothetical *tolC* targeting PNA are also given to describe why it was not chosen.

	Target	Off target
NDM1 KPN1	AcrA	None
	Fnr	Quinone oxidoreductase 2
	CsgD	None
	RecA	None
	TolC	HTH-type transcriptional regulator DmlR<br>Maltose regulon periplasmic protein<br>Citrate lyase acyl carrier protein<br>Uncharacterized hypothetical protein
CRE *E. coli*	AcrA	Bacterial leucyl aminopeptidase precursor
	Fnr	None
	CsgD	Serine endoprotease DegS<br>Type II secretion system protein D precursor
	RecA	Nucleoside diphosphate kinase ndk<br>Uncharacterized hypothetical protein
	TolC	Glutamate synthase gltD
ESBL KPN1	AcrA	None
	Fnr	Quinone oxidoreductase 2
	CsgD	None
	RecA	None
	TolC	HTH-type transcriptional regulator DmlR<br>Maltose regulon periplasmic protein<br>Citrate lyase acyl carrier protein<br>Uncharacterized hypothetical protein
MDR *E. coli*	AcrA	None
	Fnr	None
	CsgD	Serine endoprotease DegS<br>Type II secretion system protein D precursor
	RecA	Uncharacterized hypothetical protein
	TolC	Glutamate synthase gltD

Supplementary References

1. Szklarczyk, D. *et al.* STRING v10: Protein-protein interaction networks, integrated over the tree of life. *Nucleic Acids Res.* **43**, D447–D452; 10.1093/nar/gku1003 (2014).

2. Keseler, I. M. *et al.* The EcoCyc database: Reflecting new knowledge about Escherichia coli K-12. *Nucleic Acids Res.* **45**, D543–D550; 10.1093/nar/gkw1003 (2017).
