# Supplementary material for: Potentiating antibiotic efficacy via perturbation of non-essential gene expression
Source: Commun Biol. 2021 Nov 5;4:1267. doi: 10.1038/s42003-021-02783-x (PMC8571399; doi:10.1038/s42003-021-02783-x)
Supplement: Supplementary file 3 — Description of Additional Supplementary Files [file 42003_2021_2783_MOESM3_ESM.pdf]

Description of Additional Supplementary Files

File name: Supplementary Data 1

Description: Source data:

Sheet “Figure 1”: Source data seen in Figure 1b & 2a

Sheet “Figure 3”: Source data seen in Figure 3b

Sheet “Figure 4”: Source data seen in Figure 4a, b, c, & d

Sheet “Figure 5”: Source data seen in Figure 5c, d, e, f, & g

Sheet “Figure 6”: Source data seen in Figure 6b, c, & d

Sheet “Synergy Data”: Source data seen in Figure S2a & b
